# New insights into flowering date in *Prunus*: fine mapping of a major QTL in sweet cherry

**DOI:** 10.1093/hr/uhac042

**Published:** 2022-02-19

**Authors:** Camille Branchereau, José Quero-García, Nathalia Helena Zaracho-Echagüe, Laurine Lambelin, Mathieu Fouché, Bénédicte Wenden, Armel Donkpegan, Loïck Le Dantec, Teresa Barreneche, David Alletru, Julien Parmentier, Elisabeth Dirlewanger

**Affiliations:** INRAE, Univ. Bordeaux, UMR Biologie du Fruit et Pathologie, 33882 Villenave d’Ornon, France; INRAE, Univ. Bordeaux, UMR Biologie du Fruit et Pathologie, 33882 Villenave d’Ornon, France; Centre for Research in Agricultural Genomics (CRAG) CSIC-IRTA-UAB-UB, Campus UAB, 08193 Bellaterra, Barcelona, Spain; IRTA, Centre de Recerca en Agrigenómica CSIC-IRTAUAB-UB, Campus UAB, Bellaterra, 08193 Barcelona, Spain; INRAE, Univ. Bordeaux, UMR Biologie du Fruit et Pathologie, 33882 Villenave d’Ornon, France; INRAE, Univ. Bordeaux, UMR Biologie du Fruit et Pathologie, 33882 Villenave d’Ornon, France; INRAE, Univ. Bordeaux, UMR Biologie du Fruit et Pathologie, 33882 Villenave d’Ornon, France; SYSAAF-Centre INRAE Val de Loire, UMR BOA, 37380 Nouzilly France; INRAE, Univ. Bordeaux, UMR Biologie du Fruit et Pathologie, 33882 Villenave d’Ornon, France; INRAE, Univ. Bordeaux, UMR Biologie du Fruit et Pathologie, 33882 Villenave d’Ornon, France; INRAE, UE 0393, Unité Expérimentale Arboricole, F-33210 Toulenne, France; INRAE, UE 0393, Unité Expérimentale Arboricole, F-33210 Toulenne, France; INRAE, Univ. Bordeaux, UMR Biologie du Fruit et Pathologie, 33882 Villenave d’Ornon, France

## Abstract

Flowering date is an important trait in *Prunus* fruit species, especially for their adaptation in a global warming context. Numerous quantitative trait loci (QTLs) have been identified and a major one was previously located on LG4. The objectives of this study were to fine-map this QTL in sweet cherry, to identify robust candidate genes by using the new sweet cherry genome sequence of the cultivar “Regina” and to define markers usable in marker-assisted selection (MAS). We performed QTL analyses on two populations derived from crosses using cultivars “Regina” and “Garnet” as parents. The first one (n = 117) was phenotyped over ten years, while the second one (n = 1386) was evaluated during three years. Kompetitive allele specific PCR (KASP) markers located within the QTL region on LG4 were developed and mapped within this region, consisting in the first fine mapping in sweet cherry. The QTL interval was narrowed from 380 kb to 68 kb and candidate genes were identified by using the genome sequence of “Regina”. Their expression was analyzed from bud dormancy period to flowering in cultivars “Regina” and “Garnet”. Several genes, such as *PavBOI-E3, PavSR45a* and *PavSAUR71*, were differentially expressed in these two cultivars and could be then considered as promising candidate genes. Two KASP markers were validated using a population derived from a cross between cultivars “Regina” and “Lapins” and two collections, including landraces and modern cultivars. Thanks to the high synteny within the *Prunus* genus, these results give new insights into the control of flowering date in *Prunus* species and pave the way for the development of molecular breeding strategies.

## Introduction

In temperate fruit tree species, flowering date (FD) is a trait of main importance and highly dependent on the climate conditions of the production area. In sweet cherry (*Prunus avium* L.), breeding strategies for crop adaptation aim at the development of early or late blooming cultivars [1]. On the one hand, late blooming cultivars are requested in cold regions in order to avoid frost damages in early spring. On the other hand, early blooming ones are preferred in warmer regions in order to avoid high temperatures during the flowering period, which could decrease the fertility of cultivated plants by reducing stigmatic receptivity, pollen germination and pollen tube growth and therefore induce low fruit set [2,3]. Moreover, as FD is at least partially correlated to maturity date [4] (harvest time), breeders generally look for a large range of FD, so that fruit ripening spreads over time. In particular, breeders seek extra-early ripening cultivars, for which fruit can reach very high prices [1].

Flowering in perennial plants is dependent on bud dormancy, an important evolution strategy to face and survive under unfavorable climatic conditions and that allows plants to grow and bloom under optimal conditions [5]. During fall and winter, when daylight and temperatures decrease, trees enter endodormancy: internal/physiological factors prevent growth even under optimal conditions. Buds exit this deep stage of dormancy only after a certain amount of low temperatures is accumulated by the plants (fulfillment of chilling requirements, CRs). Following the endodormancy release, in late winter and beginning of spring, trees enter ecodormancy, which implies control by external/environmental factors, such as temperature and photoperiod. Bud development is prevented until optimal conditions are met later in spring and trees accumulate a sufficient amount of warm temperatures (fulfillment of heat requirements, HRs) to overcome dormancy. Flowering can finally occur. CRs and HRs estimations are time-consuming since many twigs need to be sampled and specialized equipment such as climatic chambers is required for experiments in forcing conditions. For these reasons, FD is often the single trait analyzed. This is particularly the case when large numbers of individuals have to be studied.

The phenological cycle of tree species is synchronized with alternating seasons and environmental conditions and therefore the succession of low and warm temperatures in winter and spring, respectively, is essential for flowering. Disruptions due to climate warming have already been noticed in temperate tree species like sweet cherry [6]. Indeed, reductions in available winter chill and the non-satisfaction of CRs can induce low fruit set, putting fruit production at risk and leading to important economic losses [6,7]. Investigating the genetic determinism of FD is therefore highly relevant in order to maintain the production in temperate climates.

To date, numerous quantitative trait locus (QTL) analyses on sweet cherry and other species belonging to *Prunus* genus have led to a better understanding of the genetics of this trait [4,8–10]. Moreover, the high genomic synteny of *Prunus* species often leads to the detection of QTLs in similar chromosomal regions [11]. It is well known now that FD is a quantitative trait with high broad sense heritability [4,8]. Moreover, several studies have showed that FD seems to be more dependent on CRs than on HRs in sweet cherry [8,12], as well as in other *Prunus* species like almond [10,13], apricot [14] and peach [15]. FD and CRs are highly correlated in *Prunus* species [7].

Although QTLs for FD were detected on all linkage groups (LGs) in sweet cherry, major loci were located on LGs 1 and 4 [4,8,9]. Using two sweet cherry F_1_ populations from crosses “Regina” × “Garnet” and “Regina” × “Lapins”, Castède et al. [8] detected major QTLs for both CRs and FD, stable over the years of evaluation, and in the same region of LG4. Such colocalizations were also found on LGs 1 and 7 of parents “Lapins” and “Regina”, respectively, and confirmed the high correlation between both traits and the importance of CRs for FD. A minor QTL for HRs was also found within the LG4 region. Cai et al. [16] identified QTLs for FD in three F_1_ populations of sour cherry (*Prunus cerasus*), among them two were located on LGs 1 and 4. This detection on sour cherry is relevant for QTL analyses in sweet cherry since half of the sour cherry genome is derived from sweet cherry, the sour cherry genome being divided in two subgenomes from two *Prunus* species, “avium” subgenome and “fruticosa” subgenome [17]. Calle et al. [9] used six sweet cherry populations (four F_1_ and two F_2_), most of them obtained from the early blooming cultivar “Cristobalina”, with low CRs [12], and detected a major QTL for FD on LG1. This locus overlapped with QTLs for CRs and FD described in Castède et al. [8].

Candidate genes have been suggested for QTLs on LGs 1 and 4 [18–20]. The QTL region on LG1 carries the *DORMANCY-ASSOCIATED MADS-box* (*DAM*) genes. These genes, six in number (*DAM1–6*), were initially studied in the non-dormant evergrowing peach mutant (evg), which presents a deletion in this region and does not cease growth to enter dormancy despite dormancy-inducing conditions [18,19]. In sweet cherry, *DAM 5* and *6* were considered to be strong candidate genes for the QTLs for FD and CRs on LG1 [20]. For the QTL on LG4, the most promising candidate genes were related to gibberellin and temperature sensing pathways [20]. These candidate genes were identified using the peach genome sequence annotation [21].

The aims of this study were i) to detect stable QTLs across numerous years for FD, ii) to fine-map the major QTL on LG4, iii) to identify candidate genes within the reduced QTL interval and characterize their expression, and iiii) to develop markers usable for breeding selection. This work should contribute to increase the efficiency of breeding programs for sweet cherry and other *Prunus* species to create new cultivars well adapted to the future climatic conditions.

## Results

### Flowering date evaluation

**Figure 1:**
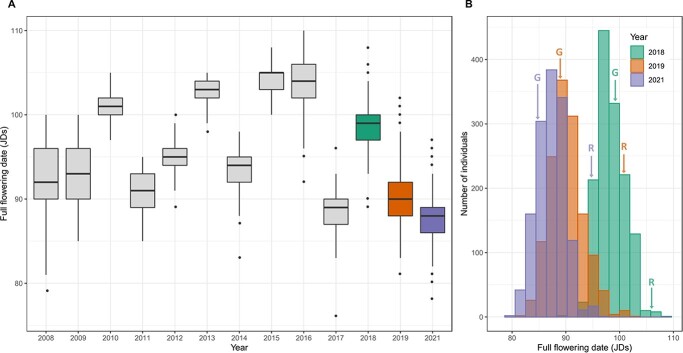
Distribution of flowering date in Population #1 (from 2008 to 2017) and Population #2 (in 2018, 2019 and 2021). **A**, box plot of full flowering distribution scored in Julian days (JDs) across ten years in Population #1 (in grey) and three years in Population #2 (2018 in green, 2019 in orange and 2021 in purple). **B**, distribution of full flowering scored in JDs across three years in Population #2 (2018 in green, 2019 in orange and 2021 in purple). Data for parental cultivars “Regina” (R) and “Garnet” (G) in 2018, 2019 and 2021 is indicated by arrows in **B**.

Populations #1 and #2 (Table 1), as well as the parental cultivars “Regina” and “Garnet”, were evaluated for three FD stages, beginning of flowering (BF), full flowering (FF) and end of flowering (EF), across several seasons from 2008 to 2021 (Fig. 1 for FF, Fig. S1 for BF and EF and Table S1) characterized by contrasted temperatures during the endodormancy and ecodormancy periods (Fig. S2 and Table S2). For instance, during the month of December (endodormancy), the mean temperatures varied from 4.6°C (season 2010–2011) to 9.1°C (season 2011–2012) whereas during the month of February (ecodormancy), the mean temperatures varied from 1.7°C (season 2011–2012) to 11.6°C (season 2020–2021) (Table S2). In 2021, EF could not be scored because of frost events in early spring. In Population #2, FDs of reciprocal crosses (R × G and G × R) were compared and no significant statistical differences were found (data not shown). Therefore, all hybrids were grouped and analyzed together in the following sections.

**Table 1:** Characteristics of the two F_1_ populations used in this study for the QTL analyses

	**Population #1**	**Population #2**
**General information**		
Cross	“Regina” × “Garnet”	“Regina” × “Garnet” (R × G) and “Garnet” × “Regina” (G × R)
Crossing method	Manual pollination	Bumblebees in confined tunnels
Year of the cross	2001	2010
Year of plantation (on own roots)	2003	2012
Number of individuals	117	1386 (793 R × G and 593 G × R)
**FD phenotyping**		
Number of years	10 years: 2008–2012 [8] and 2013–2017 new phenotyping	3 years: 2018, 2019 and 2021
Flowering stages scored	Beginning, Full and End (BF, FF and EF)	Beginning, Full and End (BF, FF and EF)
Number of phenotyped individuals	117	1386
**Genotyping**		
SNP array size (RosBREED SNP chips)	6 K [8,22]	6 + 9 K [23]
Number of genotyped individuals	117	454

FD was highly dependent on the year of evaluation (Fig. 1). In Population #1, years of evaluation could be classified into distinct groups with significant differences (results of the statistical tests not shown) of FD: 2017, 2011, 2008–2009-2014, 2012, 2010, 2013–2015-2016, from early to late FD (Fig. 1A). In Population #2, FD was significantly different across the three years of evaluation (Fig. 1). The average monthly temperatures across the seven years with early FD (2008, 2009, 2011, 2014, 2017, 2019 and 2021) were: 13.3°C in October, 8.9°C in November, 6.1°C in December, 5.7°C in January and 8.7°C in February. Across the six years with late FD (2010, 2012, 2013, 2015, 2016 and 2018), the average monthly temperatures were: 14.4°C in October, 10.9°C in November, 7.5°C in December, 6.5°C in January and 5.2°C in February. Hence, flowering occurred earlier when temperatures were lower from October to January and higher in February.

Every season, “Garnet” was the first parent to bloom. For instance, in 2018 and 2019, FF for “Garnet” was respectively seven and thirteen days earlier than “Regina” (“Garnet”: 99 and 88 Julian Days (JDs, i.e. number of days from January 1^st^), “Regina”: 106 and 101 JDs) (Fig. 1B).

Flowering stages (BF, FF and EF) were highly correlated in both populations (from 0.59 to 0.97 in Population #1 and from 0.66 to 0.94 in Population #2) (Table S3). Correlations were higher between different flowering stages within a single year (for instance in Population #2, from 0.75 to 0.80 in 2018, from 0.80 to 0.90 in 2019, and 0.94 in 2021) than for a single stage across years (from 0.76 to 0.78 for BF, from 0.68 to 0.71 for FF, and 0.71 EF).

We calculated broad-sense heritability (H^2^) for each flowering stage in both populations. In Population #1, H^2^ was equal to 0.96 for BF, FF and EF. In Population #2, heritabilities were lower: 0.90 for BF, 0.88 for FF (both calculated using three years of measurements) and 0.77 for EF (calculated with two years of measurements).

### Linkage maps

New genetic maps of “Regina” and “Garnet” were constructed using a subset of Population #2 (454 R × G hybrids). After filtering SNPs according to their quality and low missing data, 1619 SNPs were retained. Among these, 598 SNPs were heterozygous in “Regina” *(<lmxll>*), 446 heterozygous in “Garnet” (*<nnxnp>*), and 575 were heterozygous in both parents (*<hkxhk>*). As we constructed parental linkage maps and not a consensus map of both parents, we did not use the heterozygous markers in both parents. Genetic maps of each parent are described in Table S5 and Fig. S3. The map obtained for “Regina” included a higher number of markers than the one for “Garnet” (598 and 446 markers respectively). However, genetic lengths of both maps were similar. The two largest LGs were the LGs 1 of both parents, called R1 for “Regina” and G1 for “Garnet”, with 78 markers covering 136 cM and 183 markers covering 167.4 cM, respectively. The average distance between markers in “Regina” and “Garnet” parental maps were equal to 1.1 and 1.7 cM, respectively. Several large gaps were also found in the maps, especially on LGs R6 (gap = 35.5 cM), G2 (32.3 cM) and G4 (31.1 cM) (Table S5).

### QTL analyses for flowering date

QTL analyses were performed for BF, FF and EF in both populations, with year-by-year and multi-year approaches. Due to the high correlation between the three flowering stages, only the QTLs for FF are presented, as in Castède et al. [8], and are thereafter called “qP-FD”.

Concerning Population #1, QTLs were found on all LGs of “Regina” and on LGs G1, G2, G3, G5, G6 and G8 of “Garnet” using year-by-year and multi-year analyses (Tables 2 and S6). Thirteen QTLs were detected with the multi-year analysis based on ten years of data (Table 2). Only the loci on LGs R4 (*qP-FD4.1^m^*), R7 (*qP-FD7.1^m^*), G1 (*qP-FD1.2^m^*) and G6 (*qP-FD6.2^m^*) explained more than 5% of the phenotypic variation in the multi-year analysis.

**Table 2:** Flowering date quantitative trait loci (QTLs) detected with multi-year analyses in Populations #1 (across ten years) and #2 (across three years)

QTL name	LG	L (cM)	CI 95% (cM)	Physicalposition (Mb)	LOD	PVE mean (%)	d mean	Nb of years where significant
**Population #1: 117 hybrids - 10 years of evaluation (2008–2017)**								
*qP-FD1.1^m^*	R1	12.8	0–32.7	0.48–11.89	18.9	4.0	−0.9	1
*qP-FD2.1^m^*	R2	29.8	27.2–32.3	26.33–27.77	19.5	3.7	−0.9	0
*qP-FD4.1^m^*	R4	20.6	19–22.2	8.99–11.12	146.1	34.3	2.9	10
*qP-FD5.1^m^*	R5	19.0	0–42.4	3.30–14.88	11.4	2.1	0.6	0
*qP-FD6.1^m^*	R6	66.4	36.4–81.7	10.96–31.64	18.7	3.4	−0.8	3
*qP-FD7.1^m^*	R7	54.6	34.7–57.6	22.16–28.17	31.9	5.7	1.1	7
*qP-FD8.1^m^*	R8	18.1	0–43.3	1.91–18.94	8.8	1.6	−0.4	0
*qP-FD1.2^m^*	G1	128.7	101.7–151.2	39.91–54.07	24.1	7.3	1.3	4
*qP-FD2.2^m^*	G2	15.3	3.8–26.7	2.59–13.18	17.2	4.7	−1.0	0
*qP-FD3.1^m^*	G3	88.8	32–100.3	6.96–29.84	12.2	3.4	0.7	0
*qP-FD5.2^m^*	G5	11.3	0–25.6	6.82–9.10	11.4	3.1	−0.8	0
*qP-FD6.2^m^*	G6	20.7	16.9–24.4	3.07–5.79	38.5	12.8	−1.9	2
*qP-FD8.2^m^*	G8	58.0	29.1–72.1	12.40–22.82	12.2	3.1	0.9	0
**Population #2: 454 hybrids - 3 years of evaluation (2018–2019-2021)**								
*qP-FD1.1^m^*	R1	26.9	21.2–32.6	9.85–11.96	21.1	3.3	1.1	3
*qP-FD2.1^m^*	R2	23.1	15.8–30.5	23.72–27.82	12.3	1.9	0.8	3
*qP-FD4.1^m^*	R4	26.9	< 0.5 cM	9.78–10.16	180.3	37.1	−3.6	3
*qP-FD5.1^m^*	R5	35.9	20.1–51.7	10.27–18.77	17.8	2.8	1.0	3
*qP-FD6.1^m^*	R6	55.1	25.2–84.9	9.59–30.27	16.2	3.5	−1.1	3
*qP-FD7.1^m^*	R7	53.8	36.2–61.6	23.74–29.42	19.7	3	−1.0	3
*qP-FD8.3^m^*	R8	60.7	53.9–67.4	20.01–25.42	14.1	2.2	−0.8	2
*qP-FD1.2^m^*	G1	155.5	150.1–160.9	49.13–50.88	20.0	6.1	−1.5	3
*qP-FD2.2^m^*	G2	33.4	0.0–84.2	0.53–33.79	9.8	2.9	−0.7	2
*qP-FD7.2^m^*	G7	8.0	0.0–17.2	0.36–12.88	7.6	2.2	0.8	2
*qP-FD8.2^m^*	G8	26.7	8.3–45.1	2.45–17.36	6.0	1.7	0.7	1

LG, linkage group; L, distance from the beginning of the chromosome to the point of maximum LOD in the interval; CI, confidence interval; Physical position of flanking markers on “Regina” v1 genome sequence in mega base pairs (Mb); LOD, logarithm of the odds ratio; PVE mean, mean value of PVE (phenotypic variance explained by the QTL in percentage of the total variation) in the multi-environment analysis; d mean, mean value of d (difference X(A) – X(B) according to the year of evaluation, where A and B are the two homozygotes at the marker loci) in the multi-environment analysis; (+/), the sign varies according to the year of evaluation); Nb of years where significant, number of years where the QTL was detected in single-year analysis. QTLs detected every year are shaded in grey.

With Population #2, QTLs were detected on almost all LGs of both parents: all LGs of “Regina” (R1 to R8) and LGs G1, G2, G3, G7 and G8 of “Garnet” (Tables 2 and S7). Eleven QTLs were significant in the multi-year analysis and most of them were stable across years in single-year analyses (shaded in grey in Tables 2 and S7). In the multi-year analysis, only the QTLs located on LGs R4 (*qP-FD4.1^m^*) and G1 (*qP-FD1.2^m^*) explained more than 5% of the phenotypic variation (Table 2). QTLs on LGs G5 and G6 (*qP-FD5.2^m^*, *qP-FD6.2^m^*) were only detected with Population #1 and QTL on LG G7 (*qP-FD7.2^m^*) was only found with Population #2.

**Figure 2:**
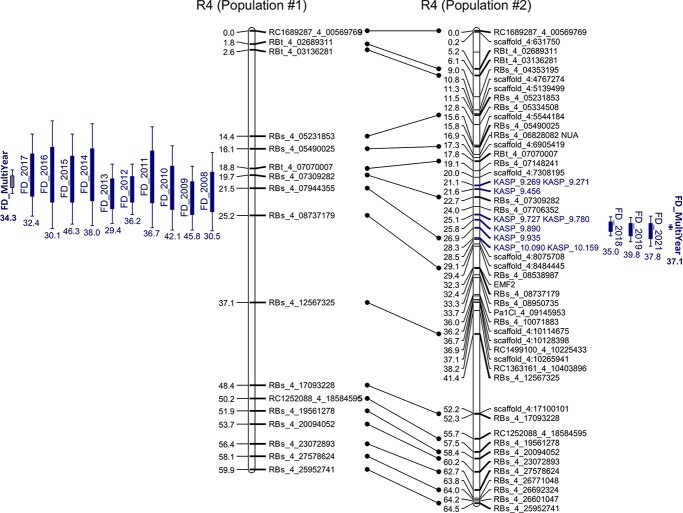
Quantitative trait loci (QTLs) for flowering date (FD) detected on “Regina” LG4 in both Populations #1 (left) and #2 (right). QTLs detected using the single-year analyses are named “FD_20xx”. QTLs detected using the multi-year analysis method are named “FD_MultiYear” and written in bold. Percentage of phenotypic variance explained (PVE) is given for each QTL. Homolog markers between both maps are linked by a line. In the map obtained with Population #2, on the right, the first set of nine KASP markers created to saturate the region of the QTL for FD and for fine mapping are colored in blue.

In both populations, the QTL on LG R4, *qP-FD4.1^m^*, explained the largest PVE (Fig. 2 and Table 2). In Population #1, it was the only locus to be significant in every single-year analysis, across the 10 years (Fig. 2 and shaded in grey in Tables 2 and S6). With the multi-year analysis, PVE were equal to 34.3% and 37.1% in Populations #1 and #2, respectively (Table 2). It reached 46.3% in 2015 in Population #1 and 39.8% in 2019 in Population #2 (Tables S6 and S7). The QTL *qP-FD4.1^m^* was detected within smaller confidence intervals in Population #2 than in Population #1: 3.2 cM in Population #1 (2 126 110 bp) and less than 0.5 cM (378 518 bp) in Population #2 (Table 2).

### Fine mapping of the QTL on LG4 of “Regina”

The fine mapping of the “Regina” LG4 region was carried out in two steps. Firstly, parental cultivars and the whole Population #2 (1386 individuals) were genotyped with nine KASP markers: KASP_9.269, KASP_9.271, KASP_9.456, KASP_9.727, KASP_9.780, KASP_9.890, KASP_9.935, KASP_10.090 and KASP_10.159 (Tables 3 and S4). These nine KASP markers were integrated in the genetic map of the LG4 of “Regina” (Fig. 2). “Garnet” was homozygous whereas “Regina” was heterozygous for the nine KASP markers. Among the 1379 individuals for which genotypes were obtained for all these KASPs, 1338 (97%) were non-recombinant: 641 were homozygous for the nine KASP markers (as observed in “Garnet”) and 697 were heterozygous for the nine KASP markers (as observed in “Regina”). The other 41 individuals were recombinant (i.e. with one recombination event between two markers) (Tables 3 and S4). The second step was to genotype these 41 recombinant individuals with eight new KASPs: KASP_9.781, KASP_9.801, KASP_9.814, KASP_9.916, KASP_9.933, KASP_9.936, KASP_9.958 and KASP_9.970 (Tables 3 and S4) to further increase fine mapping accuracy. Seventeen KASP markers were therefore used for fine mapping.

**Table 3:** Genotypes at the 17 KASPs and phenotypes (least-square mean values for Full flowering, FF, in Julian Days) of the individuals of Population #2

**KASP**	**KASP_** **9.269**	**KASP_** **9.271**	**KASP_** **9.456**	**KASP_** **9.727**	**KASP_** **9.780**	**KASP_** **9.781**	**KASP_** **9.801**	**KASP_** **9.814**	**KASP_** **9.890**	**KASP_** **9.916**	**KASP_** **9.933**	**KASP_** **9.935**	**KASP_** **9.936**	**KASP_** **9.958**	**KASP_** **9.970**	**KASP_** **10.090**	**KASP_** **10.159**	FD	Nb of ind.	Statistical significance of the difference
Position(kb)	9269	9271	9456	9727	9780	9781	9801	9814	9890	9916	9933	9935	9936	9958	9970	10 090	10 159	FF lsmeans		
**G**	T/T	A/A	A/A	A/A	A/A	C/C	A/A	G/G	C/C	G/G	C/C	G/G	A/A	C/C	A/A	A/A	T/T	90.7	G	-
**R**	C/T	G/A	A/T	A/G	A/G	C/G	A/G	G/A	A/C	G/A	C/T	A/G	A/G	C/T	A/T	A/G	T/C	100.7	R	
Non-rec.Like G	T/T	A/A	A/A	A/A	A/A	C/C	A/A	G/G	C/C	G/G	C/C	G/G	A/A	C/C	A/A	A/A	T/T	90.5	641	*** (<2.2^e^-16)
Non-rec.Like R	C/T	G/A	A/T	A/G	A/G	C/G	A/G	G/A	A/C	G/A	C/T	A/G	A/G	C/T	A/T	A/G	T/C	93.6	697	
Rec #1	C/T	G/A	A/A	A/A	A/A	C/C	A/A	G/G	C/C	G/G	C/C	G/G	A/A	C/C	A/A	A/A	T/T	91.8	2	-
Rec #2	T/T	A/A	A/T	A/G	A/G	C/G	A/G	G/A	A/C	G/A	C/T	A/G	A/G	C/T	A/T	A/G	T/C	92	1	
Rec #3	C/T	G/A	A/T	A/A	A/A	C/C	A/A	G/G	C/C	G/G	C/C	G/G	A/A	C/C	A/A	A/A	T/T	90.8	10	** (0.009396)
Rec #4	T/T	A/A	A/A	A/G	A/G	C/G	A/G	G/A	A/C	G/A	C/T	A/G	A/G	C/T	A/T	A/G	T/C	93.5	7	
Rec #5	T/T	A/A	A/A	A/A	A/A	C/C	A/G	G/A	A/C	G/A	C/T	A/G	A/G	C/T	A/T	A/G	T/C	92	1	-
Rec #6	C/T	G/A	A/T	A/G	A/G	C/G	A/G	G/A	C/C	G/G	C/C	G/G	A/A	C/C	A/A	A/A	T/T	90.7	4	NS
Rec #7	T/T	A/A	A/A	A/A	A/A	C/C	A/A	G/G	A/C	G/A	C/T	A/G	A/G	C/T	A/T	A/G	T/C	91.5	4	
Rec #8	C/T	G/A	A/T	A/G	A/G	C/G	A/G	G/A	A/C	G/G	C/C	G/G	A/A	C/C	A/A	A/A	T/T	91.3	3	* (0.03725)
Rec #9	T/T	A/A	A/A	A/A	A/A	C/C	A/A	G/G	C/C	G/A	C/T	A/G	A/G	C/T	A/T	A/G	T/C	94.1	6	
Rec #10	C/T	G/A	A/T	A/G	A/G	C/G	A/G	G/A	A/C	G/A	C/T	G/G	A/A	C/C	A/A	A/A	T/T	91	1	
Rec #11	C/T	G/A	A/T	A/G	A/G	C/G	A/G	G/A	A/C	G/A	C/T	A/G	A/G	C/C	A/A	A/A	T/T	92.7	1	-
Rec #12	C/T	G/A	A/T	A/G	A/G	C/G	A/G	G/A	A/C	G/A	C/T	A/G	A/G	C/T	A/T	A/A	T/T	94.7	1	

FF lsmeans values are presented for the parental cultivars ‘Garnet’ (G) and ‘Regina’ (R), the non-recombinant individuals (Non-rec. Like G and Non-rec. Like R) and the recombinant genotypes (Rec #1 to Rec #12). The number of individuals is presented in column ‘Nb of ind.”. The physical position of the seventeen KASP markers on the ‘Regina’ genome is given in line ‘Position (kb)’ in kilo base pairs (kb). ‘Garnet’ is homozygous for all markers, ‘Regina’ is heterozygous for all markers. ***, p < 0.001; **, p < 0.01; *, p < 0.05; NS, p > 0.05; −, not enough individuals to perform reliable statistical test. The region surrounded in black indicates the fine-mapped region.

Recombinant individuals were grouped into 12 distinct recombinant genotypes (called Rec #1 to Rec #12) depending on the position of the recombination events (Tables 3 and S8). No recombination event occurred between several markers: KASP_9.269 and KASP_9.271; KASP_9.727 and KASP_9.781; KASP_9.801 and KASP_9.814; KASP_9.916 and KASP_9.933; KASP_9.935 and KASP_9.936; KASP_9.958 and KASP_9.970; and KASP_10.090 and KASP_10.159 (Table 3). Genotypes at the 17 KASPs and least square means (lsmeans) for FD for the parents, the non-recombinant genotypes and the twelve recombinant genotypes are presented in Table 3. FD values in 2018, 2019 and 2021 of the individuals of Population #2 are presented in Table S8.

Non-recombinant individuals homozygous for all the 17 KASP markers, as “Garnet”, presented a FD similar to “Garnet” itself, with FF lsmeans equal to 90.5 and 90.7 JDs, respectively (Table 3). In comparison, the difference between FD of the non-recombinant individuals heterozygous for the 17 KASP markers, as “Regina”, and “Regina” itself, was much more important: in average, seven days of difference were found (FF lsmeans equal to 93.6 and 100.7 JDs for hybrids and “Regina”, respectively) (Table 3). “Regina” was flowering much later than the hybrids with the same non-recombinant genotype. The difference between the FDs of non-recombinant individuals as “Garnet” and of the non-recombinant individuals as “Regina” was rather small, only three days on average, but statistically significant (90.5 and 93.6 JDs, Table 3).

Among the 41 recombinant individuals, eight presented intermediate phenotypes that could not be clearly assigned to early or late flowering classes and consequently could not be used to fine-map the QTL (Table 3). These individuals belonged to therecombination groups Rec #1 (two individuals), #2 (one), #5 (one) and #7 (four). Therefore, only the remaining 33 individuals recombining between KASP_9.456 and KASP_10.090 (Rec #3, #4, #6, #8, #9, #10, #11 and #12, Table 3) were used for the fine mapping.

Seventeen individuals with Rec #3 and Rec #4 genotypes indicated that the QTL was located downstream (after) KASP_9.456: individuals that were homozygous from KASP_9.727 to KASP_10.159 (Rec #3) were early flowering while individuals that were heterozygous (Rec #4) were late flowering. FDs of these two recombinant groups (Rec#3 with 10 individuals and Rec#4 with 7 individuals) were statistically different (p < 0.01, Table 3). Thirteen individuals with Rec #6 genotype (early flowering and homozygous from KASP_9.890 to KASP_10.159), Rec #8 genotype (early flowering and homozygous from KASP_9.916 to KASP_10.159) and Rec #9 genotype (late flowering and heterozygous from KASP_9.916 to KASP_10.159) indicated that the causal region was located downstream KASP_9.890. FDs of recombinant groups Rec#8 (three individuals) and Rec#9 (six individuals) were statistically different (p < 0.05, Table 3). Based on 30 individuals, these results indicated that the QTL was located downstream KASP_9.890. A single individual with Rec #10 genotype indicated that the QTL was located downstream KASP_9.935. On the other side, one individual with Rec#11 genotype indicated that the QTL was located upstream (before) KASP_9.958.

Taken all together, these results indicate that the QTL is located between KASP_9.890 and KASP_9.958, within a region of 68 kb (Table 3). FD of individuals that were homozygous in this interval (16 individuals with Rec#1, 3 and 6) was significantly different from FD of individuals that were heterozygous (14 individuals with Rec#2, 4, 5, 7 and 12) (p-value = 0.0087).

### Identification of candidate genes for the QTL on LG4

According to the QTL analysis, the major QTL *qP-FD4.1^m^* on the LG R4 mapped between markers KASP_9.780 (9 780 346 bp) and KASP_10.159 (10 158 864 bp). We identified 65 predicted genes within this region of less than 380 000 bp (Table S9). Using transcriptomic data obtained with RNA-sequencing for the cultivars “Regina” and “Garnet” during bud dormancy over two seasons (2009/2010 and 2015/2016) [24], we found that 19 out of the 65 genes were not expressed in our plant material. The 46 remaining genes were expressed with different patterns throughout dormancy (Table S9, Fig. S4).

**Figure 3:**
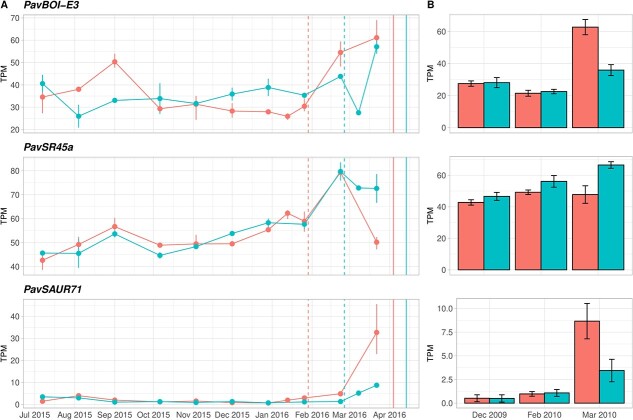
Expression profiles of three candidate genes of interest within the reduced interval of the flowering date (FD) QTL on LG4: *PavBOI-E3*, *PavSR45a* and *PavSAUR71*. Profiles are from the 2015/2016 RNA-seq analysis [24] (A) and the 2009/2010 RNA-seq analysis (B). Expression levels were measured in transcripts per million (TPM) in parental cultivars “Regina” in blue and “Garnet” in red. In A, vertical dashed and solid lines correspond to the dormancy release dates and beginning of flowering dates, respectively, for “Regina” (in blue) and “Garnet” (in red).

Based on the fine mapping, the QTL region could be redefined into a smaller region between KASP_9.890 (9 889 761 bp) and KASP_9.958 (9 957 756 bp), covering around 68 kb. Twelve candidate genes were located within this new interval (shaded in grey in Table S9). Using transcriptomic analyses, we looked for genes that were differentially expressed in “Regina” and “Garnet” during endodormancy and ecodormancy, and that could explain the FD phenotypic differences we observed in these cultivars. Among the twelve candidate genes, three exhibited differences between the two cultivars in their expression profiles after endodormancy release. These candidate genes were predicted to encode the BOI-related E3 ubiquitin-protein ligase 3 (PAV04_REGINAg0203291), the serine/arginine-rich splicing factor SR45a (PAV04_ REGINAg0203371) and the small auxin-up RNA (SAUR) protein SAUR71 (PAV04_ REGINAg0203401). We renamed them *PavBOI-E3*, *PavSR45a* and *PavSAUR71*, respectively (Fig. 3).

*PavBOI-E3* was slightly more expressed in “Regina” than in “Garnet” during endodormancy of both cultivars (prior February). However, after “Garnet” dormancy release, expression of this gene significantly increased in this cultivar. After the dormancy release of “Regina”, the gene remained more expressed in “Garnet”. Levels of expression of *PavSR45a* increased during dormancy and after “Garnet” dormancy release in both cultivars, and decreased in both cultivars after “Regina” dormancy release. The expression decline was more important in “Garnet”. *PavSAUR71* was not expressed during endodormancy and its expression started to increase after dormancy release in both parental cultivars, the increase being more important in “Garnet”. For these three candidate genes, expression profiles obtained in both RNA-seq analyses were consistent (i.e. same patterns between December and March, period in common in both analyses).

### Validation of two KASP markers

Four KASP markers (KASP_9.814, KASP_9.916, KASP_9.936 and KASP_9.958) were used to genotype an F_1_ population derived from the cross between cultivars “Regina” and “Lapins” (R × L, n = 115), accessions from the germplasm collection (n = 104) and a set of cultivars (n = 51) (Table S10).

Markers KASP_9.814 and KASP_9.916 presented a very low level of polymorphism in the two sets of accessions. In the germplasm collection, 92 accessions out of 104 were homozygous “G:G” (as “Garnet”) for KASP_9.814, and 100 were homozygous “G:G” (as “Garnet”) for KASP_9.916. In the set of 51 cultivars, 49 were homozygous “G:G” for KASP_9.814 and all were homozygous “G:G” for KASP_9.814. Therefore, we could not use them to conduct statistical analyses. For this reason, only the results obtained with KASP markers KASP_9.936 and KASP_9.958 are presented (Table 4).

**Table 4:** Allelic frequency, phenotyping data and statistical analyses for two KASP markers in the “Regina” **×** “Lapins” population, a germplasm collection and a set of cultivars

		**“Regina” × “Lapins” (n = 115)**		**Germplasm collection (n = 104)**		**Cultivars (n = 51)**	
		Nb ind.	Average FD(BF lsmeans)	Nb ind.	Average FD(BF lsmeans)	Nb ind.	Average FD(BF lsmeans)
KASP_9.936	Genotype A:A (as “Garnet”)	55	92.0	45	91.1	19	87.0
	Genotype G:A (as “Regina”)	59	94.9	46	92.7	30	88.3
	Genotype G:G	*NA*	*NA*	13	89.8	2	88.9
	Heterozygous effect(in number of days)	+ 2.9 days		+ 1.7 days		+ 1.3 days	
	P-value	< 2.2e-16^***^		2.45e-05^***^		0.02790^*^	
KASP_9.958	Genotype C:C (as “Garnet”)	55	91.9	48	91.1	21	86.6
	Genotype T:C (as “Regina”)	60	94.9	49	92.4	28	88.8
	Genotype T:T	*NA*	*NA*	7	89.7	2	88.9
	Heterozygous effect (in number of days)	+ 3.0 days		+ 1.3 days		+ 2.2 days	
	P-value	< 2.2e-16^***^		0.00101^**^		0.00015^***^	
Haplotype defined by KASP_9.936 and KASP_9.958	A:A-C:C	55	91.9	45	91.1	19	87.0
	G:A-T:C	59	94.9	43	92.7	28	88.8
	Heterozygous effect (in number of days)	+ 2.9 days		+ 1.7 days		+ 1.8 days	
	P-value	< 2.2e-16^***^		1.88e-05^***^		0.00266^**^	

In the R × L population, heterozygous individuals (52% of the population) were flowering 2.9 and 3.0 days later than homozygous ones (48%) for KASP_9.936 and KASP_9.958, respectively (p-values <2.2e-16) (Table 4). When considering the haplotypes defined by the two markers, heterozygous individuals for both markers (59 hybrids G:A for KASP_9.936 and T:C for KASP_9.958) were flowering 2.9 days later than homozygous individuals for both markers (55 hybrids A:A for KASP_9.936 and C:C for KASP_9.958) (Table 4).

In both germplasm collection and set of cultivars, a few individuals presented a new homozygous genotype (G:G for KASP_9.936 and T:T for KASP_9.958) (Table 4). Due to the low number of these individuals, we did not take them into account for the statistical analyses.

Concerning the germplasm collection, for KASP_9.936, 43% of the individuals were heterozygous and 44% were homozygous as “Garnet” (A:A) (Table 4). For KASP_9.958, 47% of the individuals were heterozygous and 46% were homozygous as “Garnet” (C:C) (Table 4). For both markers, heterozygous individuals were significantly later flowering than individuals with the “Garnet” homozygous genotype (+ 1.7 days for KASP_9.936 and + 1.3 days for KASP_9.958) (Table 4). When considering the haplotypes defined by the two KASP markers, heterozygous individuals for both markers were flowering 1.7 days later than homozygous individuals for both markers.

Concerning the panel of 51 cultivars, most individuals presented the same genotype as “Regina” and “Garnet” as well. For KASP_9.936, 59% of the individuals were heterozygous and 37% were homozygous as “Garnet” (A:A). For KASP_9.958, the heterozygous/homozygous distribution was 55% and 41% of the individuals (Table 4). Again, significant differences were observed between those individuals for KASP_9.936 (1.3 days) and KASP_9.958 (2.2 days). Significant differences were also observed when considering the haplotypes defined by both KASP_9.936 and KASP_9.958, heterozygous individuals flowering 1.8 days later than homozygous ones (Table 4).

In conclusion, for both KASP markers, significant differences were observed between heterozygous individuals as “Regina” and homozygous individuals as “Garnet” in all three panels.

## Discussion

### Flowering date evaluation and heritabilities

Within both populations, FD was not stable across years. This trait is highly dependent on temperatures and can show important inter-annual variations, resulting from the variability of the chilling and heat accumulations across years. It is known that advances in FD can be explained by an early fulfillment of the HRs induced by high temperatures in late winter [8]. In our experiments, flowering occurred earlier when temperatures from October to January were low and temperatures in February were significantly superior to the mean. This could be related to a better fulfilment of the CRs in late autumn and winter, and of the HRs in late winter. Correlations between the three stages of FD and between years were high in both populations. High heritability values were found, in the same range as those estimated in sweet cherry prior to this study [4,8,9]. Values were higher within Population #1 compared to Population #2. Data is more reproducible over ten years, reducing the effect of the environment and therefore increasing heritability.

### Linkage maps were improved with the RosBREED cherry 6 + 9 K SNP array

The linkage maps developed using the RosBREED 6 + 9 K SNP array were compared with the ones previously obtained with the RosBREED 6 K SNP array [8]. Sizes of LGs in both maps were close and no marker rearrangements were observed. The new maps contained many more markers (around four and three times more for “Regina” and “Garnet” parental maps, respectively) and average distance between markers and largest gaps were reduced. In general, LGs of “Regina” (R1 to R8) were denser than LGs of “Garnet” (G1 to G8), with lower average distance between markers. This result seems to be in accordance with the fact that “Regina” is highly heterozygous compared to other modern cultivars [25]. Although using a larger number of markers allowed filling several gaps found in maps obtained with the RosBREED 6 K SNP array [8], some large gaps remained on LGs R6, G2 and G4, which may be caused by a lack of recombination events within these regions in the cultivars.

Recently, Calle et al. [26] presented parental linkage maps of cultivars “Vic” and “Cristobalina” using the RosBREED 6 + 9 K SNP array and a 161-individuals F_1_ population. These maps respectively contained 910 SNPs covering 636.7 cM and 789 SNPs covering 666.0 cM. However, only 324 and 310 SNP markers mapped at unique genetic positions in “Vic” and “Cristobalina” parental maps, respectively [26]. In our study, the genotyping of 454 individuals with the RosBREED cherry 6 + 9 K array allowed to develop maps that contained more markers mapping at unique positions (459 out of 598 for the “Regina” parental map and 330 out of 446 for the “Garnet” parental map) and that were slightly shorter than those developed by Calle et al. [26]. Therefore, the maps we developed present a higher marker density. This can be due to the number of individuals: we increased the probability of recombination events by using more individuals, leading to the development of genetic maps with a reduced average distance between markers.

### Genetic determinism of flowering date

Few QTLs for FD were detected within Population #1 with the single-year analyses, three on average, which could be related to the reduced size of the population. In comparison, more QTLs were detected with the single-year analyses within Population #2, nine on average, and confidence intervals were smaller. The detection was highly improved in Population #1 when combining all years together through the multi-year analysis. QTLs were detected on almost all LGs (thirteen), with some of them accounting for a very small proportion of the phenotypic variance, and confidence intervals of the major QTLs detected with the single-year analysis were reduced. Prior to our study, Castède et al. [8] performed multi-year analysis on Population #1 with five years of measurements and detected QTLs on eleven LGs. Here, the addition of five years of FD measurements led to a significant reduction in the size of the QTLs confidence intervals and the detection of new minor QTLs (*qP-FD5.1^m^* and *qP-FD8.1^m^*). With Population #2, confidence intervals were further reduced with the multi-year detection analysis. Working with a population containing more individuals increased the power and the accuracy of the detection. QTL analyses were very consistent in both populations: the large majority of the loci detected with Population #1 were significant with Population #2 as well. The QTL *qP-FD6.2^m^* on LG G6 was an exception. This QTL was detected with Population #1 across a few years and showed PVE values up to 14.6%, while it was not found in any of the analyses performed with Population #2. Because it was not stable across years, the significance of this QTL could be due to specific climatic conditions and/or genotype × environment interactions (in 2011 and 2016), which were not found in the three years of measurements in Population #2.

Overall, the large number of QTLs detected confirmed that FD is a complex trait with a polygenic control. Most of these QTLs were also found in peach and apricot [4]. QTLs on LGs G1 (*qP-FD1.2^m^*) and R4 (*qP-FD4.1^m^*) were the only loci with PVE values higher than 5% to be found in common in both populations. Both were firstly identified in sweet cherry by Dirlewanger et al. [4] and have also been reported in other *Prunus* species including peach, apricot, almond and sour cherry [10,13,16,27]. The QTL on LG1 was detected in “Lapins” and “Garnet” using a “Regina” × “Lapins” (R × L) population [4,8] and Population #1 [8]. In both cultivars, the QTL mapped at the bottom of LG1. More recently, Calle et al. [9] detected a major QTL (PVE up to 60.9%) at the bottom of the LG1 using several populations derived from the extra-early blooming parental cultivar “Cristobalina” [12]. In our study, the QTL on LG1 of “Garnet” showed PVE values up to 17.1% in Population #1 (FF 2008), 8.8% in Population #2 (FF 2018) and mapped at the end of the LG. Confidence intervals of this QTL colocalize in “Garnet”, “Lapins” and “Cristobalina” and cover the chromosomal region known to carry *DAM* genes [9,18,20,28].

The QTL that explained the highest percentage of phenotypic variation within both populations was *qP-FD4.1^m^* on LG R4. The high significance of this locus was demonstrated in Dirlewanger et al. [4] and later in Castède et al. [8] using both R × L population and Population #1 in which it explained up to 31.8% and 45.8% of the phenotypic variation of FF, respectively. Our study also demonstrated the high stability of *qP-FD4.1^m^*: this QTL alone was significant across all the growing seasons. In both single and multi-year analyses, the accuracy of the analyses for this major QTL was significantly improved with Population #2. While the QTL region spanned 2 126 110 bp in Population #1 (multi-year analysis), it covered around 380 000 bp in Population #2, consisting in a reduction of more than 1.7 Mb. This result could be also explained by the denser genetic maps of the R × G Population #2 (obtained with the RosBREED cherry 6 + 9 K SNP array) as compared to those of Population #1 (obtained with the RosBREED cherry 6 K SNP array) and by KASP markers added in the region of the QTL.

Our study is the first one presenting QTL analyses using two different experimental designs obtained from a same cross, “Regina” × “Garnet”. It provides important information from a methodological point of view, as we were able to compare QTL analyses performed on a small population of 117 individuals evaluated for FD during ten years (Population #1) and a large population of 454 individuals evaluated for FD during three years (Population #2). When using a small population, single-year QTL analyses allowed to detect few QTLs within large confidence intervals. Multi-year analyses improved detections; however, as showed when comparing our results with those presented by Castède et al. [8], a high number of years of phenotyping is required. In comparison, many more QTLs were detected in single-year analyses when using a large population. This is particularly interesting for minor QTLs. Moreover, QTL confidence intervals were reduced. Multi-year analyses further enhance the accuracy of the QTL detections, even if only a few years of phenotyping (three years for Population #2) are available. In the present study, using a large population significantly improved the QTL analyses. However, two different SNP arrays were used to genotype Populations #1 and #2, therefore, genotyping is also of main importance. Our study showed that using a large number of individuals genotyped with a large amount of markers leads to more accurate QTLs. This strategy is particularly adapted to the study of agronomical traits that can be quickly scored, such as FD. However, since the period covered by the flowering of a sweet cherry population is in general less than four weeks, phenotyping might become challenging if several thousands of progenies were to be scored.

### Fine mapping of *qP-FD4.1^m^*, a major flowering date QTL on LG4

The first objective of fine mapping is to narrow a given QTL region in order to reduce the number of candidate genes. This strategy has already been used in peach for several traits, among them fruit acidity [29] (fine mapping of the *D* locus within a 100 kb region), maturity date [30] (fine mapping of the major LG4 locus in a 220 kb interval), plant height [31] (fine mapping of the *Tssd* gene in a 500 kb region), powdery mildew resistance [32] (fine mapping of the *Vrn3* gene in a 270 kb region) and skin fuzziness [33] (fine mapping of the *G* locus in a 481 kb interval).

In our study, we aimed to precisely map the sweet cherry FD QTL *qP-FD4.1^m^* that we detected in a 380 kb-interval on LG4 with MultiQTL, between markers KASP_9.780 and KASP_10.159. With the fine mapping, we concluded that it was located between KASP_9.890 and KASP_9.958, within a 68 kb-region. Developing tightly linked KASP markers and phenotyping the whole Population #2 allowed us to improve the QTL localization. However, we must remain cautious and take into consideration several limits in our experiments. First of all, the phenotypic differences were small. Although “Regina” and “Garnet” were well differentiated, late and early flowering respectively, hybrids with non-recombinant parental genotypes differed by only three days on average. Small FD differences were observed between our recombinant genotypes as well. The whole population tended to flower rather early, closer to “Garnet” than “Regina”. Unlike other important agronomic traits in peach from studies cited earlier, FD is not controlled by only one major gene or QTL. Therefore, phenotypic variations which are non-related to the QTL on LG4 may occur in our recombinants. This could be the case for example for recombinant genotypes #1, #2, #5 and #7, which did not allow to precise the QTL position. Finally, we only had 41 individuals with recombinant genotypes, among which 33 with contrasted phenotypes could be used to fine-map the QTL. The mapping resolution depends on the number of recombinants and even with a large effect QTL, the higher the number of recombinants, the better the mapping accuracy.

Fine mapping of this FD QTL in sweet cherry was of main interest, in particular because LG4 is considered to be a hot spot QTL LG in sweet cherry. Indeed, it is known to carry major QTLs associated to fruit firmness [34], maturity date [35] and rain-induced fruit cracking [36]. All these QTLs map at distinct but close positions on the same LG. Therefore, precising their location as much as possible would allow breeders to optimize marker-assisted selection (MAS) for different key agronomical traits simultaneously.

### Candidate genes for flowering date within the QTL on LG4

While several studies have confirmed that the QTL for FD on LG1 contains *DAM* genes [18–20,27,37], little is known about the QTL on LG4. In almond, the major gene *Late blooming* (*Lb*) was identified in LG4 [38] but no candidate genes co-localizing with this locus have been successfully found [39]. In sweet cherry, to date, the most promising candidate genes are related to gibberellins and temperature sensing pathways [20]. They were identified by using the peach genome sequence annotation.

In our study, we used the “Regina” genome sequence and identified 65 new candidate genes, among which 46 were expressed in our plant material. QTL analyses with Population #2 allowed us to reduce the size of the QTL interval and none of the candidate genes selected in Castède et al. [20] mapped within this refined region. Two of them, *EMF2* (*EMBRYONIC FLOWER2*) and *NUA* (*NUCLEAR PORE ANCHOR*), were included in the new map and were found far from our QTL region (Fig. 2).

We could reduce our 46 candidate genes list down to a set of twelve genes based on fine mapping. Several genes, differentially expressed in “Regina” and “Garnet”, could be considered as promising, such as *PavBOI-E3, PavSR45a* and *PavSAUR71*. In Arabidopsis, the RING domain E3 ligase BOI represses flowering by repressing the expression of *FLOWERING LOCUS T* (*FT*) by two different ways: BOI binds to CONSTANS (CO) to inhibit its targeting to *FT*; or BOI targets *FT* via DELLA proteins [40]. Both mechanisms result in decreased expression of *FT* mRNA and inhibit flowering. Based on our transcriptomic experiments, *PavBOI-E3* was more expressed in “Regina” than in “Garnet” during endodormancy. This could be related to a stronger flowering inhibition in “Regina”. However, after “Garnet” dormancy release, *PavBOI-E3* expression importantly increased in “Garnet” and became higher than in “Regina”, which is not anymore in accordance with the predicted function of the gene and its effect on FD. *PavSR45a* was also differentially expressed between “Regina” and “Garnet” especially after the endodormancy release period. This gene encodes the serine/arginine-rich (SR) protein SR45a, a splicing factor (*PavSR45a*). In *Arabidopsis thaliana*, the loss-of-function mutant *sr45–1* exhibits pleiotropic phenotypes, among them a late flowering phenotype and elevated levels of FLOWERING LOCUS C (FLC), a major flowering repressor [41] within the Brassicaceae family. It was demonstrated that SR45 influences the autonomous flowering pathway in a FLC-dependent way in Arabidopsis [41,42]. SR45 has also been related to the epigenetic regulation of *FLOWERING WAGENINGEN* (*FWA*), another flowering-related gene [43]. SR45 protein was also reported to affect the alternative splicing of other SR genes [41] and to negatively regulate sugar signaling by repressing glucose-induced ABA accumulation [44]. Therefore, SR45 is an important splicing factor regulating genes involved in growth, development and response to environmental changes. In our material, expression levels of this gene increased during dormancy, reached a peak and then decreased after “Regina” dormancy release in both cultivars. *PavSR45a* was slightly more expressed in “Regina”, especially during the late sampling dates of both transcriptomic analyses. Moreover, the expression drop was faster in “Garnet”. We could hypothesize that several flowering-related genes are down-regulated by SR45. Hence, the lower expression level observed in “Garnet” could lead to an earlier flowering as compared to “Regina”. *PavSAUR71* is also a promising CG. *SAUR* genes constitute the largest family of early auxin-responsive genes and play crucial roles in plant growth and development control [45]. In both “Regina” and “Garnet” cultivars, expression of this gene was null during endodormancy and started to increase after dormancy release. This is in accordance with increases of auxin levels during dormancy release reported in several studies [46]. *PavSAUR71* expression levels increased more importantly in “Garnet” and could be related to its early flowering phenotype, compared to “Regina”. Several genes encoded a G-type lectin S-receptor-like serine threonine-kinase (GsSRK) that regulates both plant architecture and salt stress responses [47]. A gene encoding a kelch-repeat domain containing F-box protein (KFB) was also found within the fine mapping interval. In Arabidopsis, several KFBs are known to be involved in circadian clock and photoperiodic flowering time regulation [48]. However, the expression profiles of this gene were not conclusive.

Although they were not located within the fine mapping confidence interval, some genes with relevant functions were worthy to consider. PAV04_REGINAg0203151 encodes the CONSTANS-LIKE 3 (COL3) transcription factor (TF) from the CCT (CO, CO-like and TOC1) family. To date, genes from the CCT family are manly described in cereal crops, where they are involved in the control of flowering time in response to the photoperiod and the circadian clock [49–51]. In trees, reductions in day length have been shown to induce growth cessation and bud dormancy, however, the molecular mechanisms underlying the effects of photoperiod on growth and developmental transitions remain quite unclear [52]. PAV04_REGINAg0203191 codes for the enzyme ABA3, the molybdenum cofactor sulfurase involved in the synthesis of the sulfureted form of the molybdenum cofactor which is required for the activity of molybdenum enzymes such as aldehyde oxidase (AO) [53]. AO catalyzes the final step of the biosynthesis of abscisic acid (ABA). Therefore, ABA3 plays an essential role in the biosynthesis of ABA, a major plant hormone promoting seed and bud dormancy [46,53]. Finally, PAV04_REGINAg0203201 encodes the JUNGBRUNNEN 1 (JUB1) TF from the NAC (NAM, ATAF, CUC) family. NAC TFs constitute one of the largest TF families in plants and are reported to participate in numerous processes including plant growth, development, stress responses and senescence [54]. In *Rosaceae* species peach, apple and sweet cherry, NAC TFs have been reported as candidate genes for a maturity date QTL located in a distinct region of LG4 [30,35,55]. In *Arabidopsis*, JUB1 TF represses *GA3ox1* and *DWF4* genes involved in gibberellins and brassinosteroids biosynthesis pathways, leading to reduced levels of these hormones and the accumulation of DELLA proteins, restricting plant growth while promoting stress tolerance [56]. JUB1 delays senescence, modulates cellular H_2_O_2_ levels and also enhances various abiotic stress tolerance responses by targeting DREB2A [57]. Recently, *ABA3*, *JUB1* and *COL3* were found to be under positive selection during apricot domestication, most likely for selection on tree phenology and environment adaptation [58].

### KASP markers usable in marker-assisted selection

For two KASP markers, KASP_9.936 and KASP_9.958, we found that the heterozygous accessions (as “Regina”) were significantly later flowering than homozygous ones (as “Garnet”) using two validation panels and a F_1_ population. Phenotypic differences between heterozygous and homozygous individuals were much more important in the R × L population (three days) than in both panels (between one and two days). It is known that the QTL on LG4 is the major locus in this population as well [4,8]. However, the germplasm collection and the panel of cultivars exhibit a much larger genetic diversity. A large number of QTLs are likely to be involved in the control of FD and the QTL on LG4 may not be the major FD QTL in this material. This could explain why the effect of this QTL was lower in these two panels. Nevertheless, this result can be useful in a MAS program.

In the diversity panels (germplasm collection and cultivars), some individuals had an additional homozygous genotype compared to the population. In the set of 51 cultivars, those individuals (two in number, that is 4% of the panel) presented a late flowering phenotype. They were slightly later flowering than heterozygous individuals for both KASPs. This was in accordance with what we expected from allele combinations: for instance for KASP_9.958, homozygous C:C are early FD, heterozygous T:C are late flowering, and homozygous T:T are further late flowering. Different results were found in the germplasm collection. Firstly, more individuals presented the new homozygous genotype (6% for KASP_9.958 and 12% for KASP_9.936), confirming that a larger genetic diversity is present in the collection. Interestingly, these individuals were flowering earlier than those having the other homozygous genotype (as “Garnet”). One explanation could be that these individuals have the early allelic combination at other FD QTLs. Moreover, in these genetic backgrounds, the QTL on LG4 might not be the one explaining the largest PVE.

FD is a quantitative trait controlled by many genomic regions. The major QTL on LG4 is one of them, but other regions such as LG1 play an important role too [9]. Therefore, markers should be designed in these other important genomic regions. Recently, Calle et al. [28] developed two DNA-based markers in the QTL on LG1, within the DAM genes regions. Developed from the extra-early cultivar “Cristobalina”, these markers could be useful for selection for early flowering and low CRs in sweet cherry. Combined together, these new markers should allow the deployment of a complete MAS strategy in sweet cherry for FD.

## Conclusion

We report in this study the first fine mapping performed in sweet cherry, for the major FD QTL on LG4. The QTL on LG4 was highly stable in our plant material, which has relatively high CRs and is rather late blooming. Therefore, this region is of main interest in sweet cherry breeding programs as well as the QTL on LG1 associated to low chilling cultivars to create new cultivars well adapted to their growing area. Our results constitute the first step for the development of a set of markers within the LG4 QTL that could be used in MAS for FD in sweet cherry. We reduced the 380-kb region obtained with the new QTL analyses using a large population to an 68-kb region containing only twelve candidate genes. The most likely candidate genes, with interesting expression patterns, were related to splicing (*SR45a*) and auxin-response (*SAUR71*). Further analyses based on transformation experiments on model species could be performed to validate the robust candidate genes we identified and might give new insights into the control of FD in *Prunus* species. Moreover, our study provides relevant information from a methodological point of view by using a same cross in two different experimental designs to compare QTL analyses.

## Materials and methods

### Plant material

Two F_1_ sweet cherry populations derived from crosses using “Regina” and “Garnet” cultivars were analyzed (Table 1). “Regina” is a late blooming German cultivar, whereas “Garnet” is an early blooming cultivar from the USA. The first population, called hereafter “Population #1”, is composed of 117 individuals obtained from the cross “Regina” × “Garnet”. Trees were planted in 2003 on their own roots (not grafted, therefore planted without replication) in the Tree Experimental Unit (UEA) of the French National Research Institute for Agriculture, Food and the Environment (INRAE)-Bordeaux research center, at Toulenne (50 km south-east from Bordeaux, France). Trees were planted every 2.5 meters in rows separated by six meters, orchards were not irrigated. This population has been firstly used and presented in Castède et al. [8]. The second population, called “Population #2”, is composed of 1386 hybrids. The cross was made in 2010 using potted trees (five trees of each parental cultivar) and bumblebees in confined tunnels. Among the 1386 hybrids created, 793 had “Regina” (R × G hybrids) while 593 had “Garnet” (G × R hybrids) as maternal parent, respectively. Population #2 was planted in 2012 in the same experimental site as Population #1. Trees were planted every two meters in rows separated by five meters.

For the KASP marker validation, a population derived from a cross using “Regina” and “Lapins” (R × L) and two sets of accessions were used (Table S10). The R × L population is composed of 115 individuals planted on their own roots in the UEA of INRAE, in Toulenne. The first set of accessions is a subset of 104 accessions from the sweet cherry core collection defined from the INRAE sweet cherry germplasm collection, maintained by the INRAE’s *Prunus* Genetic Resources Center in Bourran (120 km south-east from Bordeaux, France) [59]. This panel is already well characterized and we carefully selected individuals representing a large genetic diversity and covering a large variability for FD [59]. The second one is a set of 51 cultivars, including modern cultivars, planted in Toulenne, in the same area as the three other populations.

For the RNA-seq analyses, flower bud samples were collected during two seasons from “Garnet” and “Regina” trees grown at the INRAE UEA in Toulenne (December 2009–March 2010) and Bourran (July 2015 – March 2016). As previously described in Vimont et al. [24], a mix of randomly chosen flower buds (equivalent to a 2 mL volume) were harvested from branches of two or three different trees, corresponding to the biological replicates. Upon harvesting, buds were flash frozen in liquid nitrogen and stored at −80°C prior to performing RNA-seq.

### Flowering date phenotyping

Three flowering stages were scored: beginning of flowering (BF), when approximately 10% of the floral buds reached full bloom; full flowering (FF), when 75% of the floral buds reached full bloom; end of flowering (EF), when more than 50% of the flowers were wilting. Trees were observed from three to four times a week during the season to score the different flowering stages in Julian days, JDs. Within Population #1, FD was evaluated during ten years from 2008 to 2017. The first five years of evaluation have been utilized in Castède et al. [8]. In the present study, five additional years from 2013 to 2017 were used to refine the QTL analyses. Within Population #2, BF and FF were evaluated during three years, in 2018, 2019 and 2021 while EF was evaluated in 2018 and 2019 but not in 2021 because of frost events in March–April preventing a reliable evaluation (Table 1).

For KASP validation, FD (BF stage) was scored during ten years in the R × L population, from 2006 to 2016 (Table S10). The two sets of accessions were evaluated for BF during six years, from 2014 to 2019, each accession being evaluated at least three years (Table S10).

Daily temperatures were collected in the orchard located at Toulenne using an automatic data-logger (Ebro®; Ebro Electronic, Ingolstadt, Germany) in order to characterize environmentally the years of evaluation.

### Measurements of bud break and estimation of the dormancy release date

Measurements for the dormancy stages were performed on randomly chosen branches cut every two weeks from November 16^th^ 2015 to April 4^th^ 2016 for “Garnet” and “Regina”. Branches were incubated in water pots placed in a growth chamber (25°C, 16 h light/8 h dark, 60–70% humidity). The water was replaced every 3–4 days. After ten days under these forcing conditions, the percentage of bud break, i.e. flower buds at BBCH stage 53 [60] was recorded. The date of dormancy release was estimated when at least 50% of the flower buds were at the BBCH stage 53 or higher after ten days under forcing conditions.

### Statistical analysis

Distribution, mean, minimum and maximum values of BF, FF and EF were estimated for each year. Additionally, Spearman correlation coefficients between the three flowering stages and between years were calculated. Analyses were performed using “ggplot2” and “psych” R packages. Broad-sense heritability (H^2^), to measure the between-year stability of the flowering traits, was also estimated from the analysis of variance based on the following mixed model, as previously described in Dirlewanger et al. [4]:

Y_ij_ = μ + g_i_ + y_j_ + e_ij_.

where Y_ij_ is the phenotypic value of the i^th^ individual in the j^th^ year, μ is the mean value of the trait, g_i_ is the random genotypic effect of individual i, y_j_ is the fixed effect of year j and e_ij_ is the residual of the model (i.e. genotype × year interaction). This linear mixed-effects model was fitted in R using the lme4 package [61].

Heritability was then estimated using the following equation:

H^2^ = $\frac{\upsigma_{\mathrm{g}}^2}{\Big({\upsigma}_{\mathrm{g}}^2+\frac{\upsigma_{\mathrm{e}}^2}{n}\ \Big)}$where ${\upsigma}_{\mathrm{g}}^2$ is the genetic variance, ${\upsigma}_{\mathrm{e}}^2$ the residual variance (environmental variance) and n is the number of years.

Heritabilities were calculated for both populations.

### Genotyping and linkage mapping

Genomic DNA from parental lines and from hybrids from Population #2 was extracted from young leaves. A subset of 454 R × G hybrids (randomly chosen) from Population #2 was genotyped with the RosBREED cherry 6 + 9 K Illumina Infinium® SNP array [23]. The strategy consisting in the genotyping of a subset of Population #2 was followed to reduce the cost of the analyses. SNP genotype analyses and marker filtering were done using GenomeStudio software (v2.0, Illumina) as described in Klagges et al. [62]. Monomorphic markers, individuals with more than 5% missing data and markers with more than 10% missing data were discarded to construct linkage maps of each parent “Regina” and “Garnet”, following the pseudo test-cross methodology used for heterozygous species [63]. JoinMap® software [64] (version 4.1) was used to perform linkage analysis using SNPs which were heterozygous in only one of the two parents (classes coded *<lm x ll >* and *< nn x np>*). The independence LOD test (threshold = 15.0) and the regression mapping function were used for markers grouping and maps construction, respectively. SNPs with identical segregation in the population were included in the maps (function “Assign identical loci to their groups”).

### QTL analyses for flowering date

QTL analyses were based on linkage maps already available for Population #1 [8] and on the new maps for Population #2. For both populations, QTL analyses were performed for the three FD stages using the Multiple Interval Mapping (MIM) method implemented in MultiQTL V2.6 software (http://www.multiqtl.com). Detections were carried out separately for “Regina” and “Garnet” parental maps by using the “single QTL model” (one-QTL per linkage group). We tested the “two-linked QTLs model” but no consistent detections were observed (results not shown). Both single-year (or year-by-year) and multi-year models were utilized (multi-environment model available in MultiQTL). When performing multi-year analysis, a single position (in cM) and a single LOD value are given while values of percentage of variation explained (PVE) are estimated for each year. For ease of reading, the mean PVE value across years is presented. This study gives an update of the QTL analyses presented in Castède et al. [8] by using five additional years of FD phenotyping to perform a multi-year analysis with the 10 years available in total, from 2008 to 2017. Graphical representation of QTLs on linkage maps was generated with MapChart software v2.3 [65]. Only QTLs for FF were presented in this paper. In accordance with to the GDR nomenclature (see www.rosaceae.org for more details), they were called “qP-FD”.

### Fine mapping of the QTL on “Regina” LG4 with KASP markers

SNPs located within the confidence interval of the FD QTL on LG4 were selected from different sources: i) SNPs within the RosBREED cherry 6 + 9 K Illumina Infinium® SNP array, based on their position on the sweet cherry physical map; ii) SNPs from GBS analyses described in a previous study [66]; and iii) SNPs identified through the mapping of available “Regina” RNA-sequencing data on the “Regina” genome sequence (Table S4). In this last case, SNPs were identified using IGV software (http://software.broadinstitute.org/software/igv/), and heterozygous markers with high RNA-seq coverage were selected. Well-distributed SNPs were then selected to be transformed into Kompetitive Allele Specific PCR (KASP) markers based on the dual Fluorescence Resonance Energy Transfer (FRET) method, in which the sample DNA is amplified with allele specific primers conjugated to fluorometric dyes HEX and FAM at their 5′ end. For each KASP, three primers were developed by the BioGEVES laboratory (Beaucouzé, France) and the reactions were performed as described in Bernard et al [67].

Fine mapping of the QTL region on LG R4 was carried out into two steps. Firstly, nine KASPs were used to genotype the complete Population #2 (1386 individuals): KASP_9.269, KASP_9.271, KASP_9.456, KASP_9.727, KASP_9.780, KASP_9.890, KASP_9.935, KASP_10.090 and KASP_10.159 (named accordingly to their physical position on sweet cherry LG4 in kb). Recombinant individuals detected in this region were then genotyped with eight new KASP markers, selected on IGV software and located within genes between KASP_9.780 and KASP_10.159, named KASP_9.781 (gene PAV04_REGINAg0203151), KASP_9.801 (gene PAV04_REGINAg0203181), KASP_9.814 (gene PAV04_REGINAg0203191), KASP_9.916 (gene PAV04_REGINAg0203361), KASP_9.933 (gene PAV04_REGINAg0203391), KASP_9.936 (gene PAV04_REGINAg0203391), KASP_9.958 (gene PAV04_REGINAg0203421) and KASP_9.970 (in the gene PAV04_REGINAg0203451). Least square means (lsmeans) of the three years of data (2018, 2019 and 2021) for FD were calculated for the parents, the non-recombinant genotypes and the groups of same recombinant genotypes. With this fine mapping of the QTL, our objective was to reduce the confidence interval of its position and, further, to reduce the number of putative candidate genes.

### *In silico* candidate genes identification

Chromosomal region for *in silico* CG analysis was selected based on the QTL on LG4 detected with MultiQTL within Population #2 (QTL analyses using 454 R × G hybrids). Predicted cherry gene models and corresponding protein sequences of this genomic region were retrieved from “Regina” sweet cherry genome repository [68] (https://doi.org/10.15454/KEW474). Data mining on the gene ontology terms associated with candidate genes was done using Blast2GO Version 1.4.4 [69]. Predicted cherry peptides were used for similarity search in a non-redundant genebank protein database with blastp algorithm with a minimum e-value <10^−6^ before gene ontology mapping and annotation. When no functional annotation from Blast2GO was available, an annotation from the Arabidopsis database TAIR (The Arabidopsis Information Resource) was added.

### RNA-seq data and analyses

Transcriptomic data was used to look for differentially expressed genes in cultivars “Regina” and “Garnet” which could potentially explain the FD differences we observed. We used the results of two experiments of whole transcriptome analyses (RNA-sequencing) performed in 2009/2010 and 2015/2016 on cultivars “Regina” and “Garnet”, in order to obtain the expression profiles of the candidate genes.

During the 2009/2010 sampling season, three dates were chosen for RNA sequencing: 3 December 2009, 1 February 2010 and 16 March 2010, associated with different stages of dormancy, respectively endodormancy, endodormancy release and ecodormancy. For both cultivars, three biological replicates were used at the three dates. Total RNA was extracted from 50 mg of frozen ground flower buds and sequenced on an Illumina® HiSeq 2000 (single read) by GATC Biotech (Mulhouse, France) in 2011. For the 2015/2016 samples, total RNA was extracted and sequenced as described in Vimont et al. [24]. Eleven dates spanning from July 2015 to the end of March 2016 were used, including the three mentioned dormancy stages covered with the 2009/2010 sampling. Sequencing data are available online (BioProject PRJNA756935 and Gene Expression Omnibus GSE130426). The quality of raw reads was assessed using FastQC (www.bioinformatics.babraham.ac.uk/projects/fastqc/) and possible adaptor contaminations and low quality trailing sequences were removed using Trimmomatic [70]. Raw reads sequences were mapped on the sweet cherry “Regina” reference genome [68] (v1.0) using STAR as previously described in Vimont et al. [71]. Raw counts and transcripts per million reads (TPM) for each transcript were calculated using HTSeq [72,73].

### KASPs validation

Four KASP markers from the set of the seventeen markers used for the fine mapping, were tested for validation on different genetic backgrounds. We selected markers among those included within the QTL interval established by our fine mapping approach, spanning from KASP_9.890 to KASP_9.958: KASP_9.916, KASP_9.936 and KASP_9.958. Moreover, although it was outside the interval, we also selected KASP_9.814 as several recombination events occurred between this marker and KASP_9.890 (eight individuals out of 41 were recombinant between these markers). Recombination events occurred between KASP_9.890 and KASP_9.916 for nine individuals, therefore, we also selected KASP_9.916. No recombination occurred between KASP_9.916 and KASP_9.933, so we did not select KASP_9.933. A recombination occurred between KASP_9.933 and KASP_9.935. However, as KASP_9.935 and KASP_9.936 were very close and no recombination event occurred between them, we decided to select KASP_9.936. Finally, one recombination event occurred between KASP_9.936 and KASP_9.958, so we selected KASP_9.958. In the end, KASP_9.814, KASP_9.916, KASP_9.936 and KASP_9.958 were tested for validation on the R × L population and on the two sets of accessions (Table S10).

In order to determine if FD was different between heterozygous and homozygous genotypes, analyses of variances (ANOVAs) were performed in R software. The following linear model was used:

Y_ij_ = μ + g_i_ + y_j_ + e_ij_ where Y_ij_ is the phenotypic value of the i^th^ individual in the j^th^ year, μ is the mean value of the trait, g_i_ is the random genotypic effect of individual i, y_j_ is the fixed effect of year j and e_ij_ is the residual of the model.

## Supplementary Material

Web_Material_uhac042

## Data Availability

Phenotypic data are available in the excel file “Raw data - Phenotyping data”. Assembly and annotation of the “Regina” sweet cherry genome is available at https://data.inrae.fr/dataset.xhtml?persistentId=doi:10.15454/KEW474 RNA-seq data from 2015-2016 have been deposited in the NCBI Gene Expression Omnibus under the accession code GSE130426. RNA-seq data from 2009–2010 have been deposited in the NCBI Short Read Archive under the accession code PRJNA756935 (https://www.ncbi.nlm.nih.gov/sra/PRJNA756935).
